# A new late Pleistocene fossil crocodile from Sudan reveals hidden diversity of *Crocodylus* in Africa

**DOI:** 10.1038/s41598-025-08980-6

**Published:** 2025-08-01

**Authors:** Khalafallah Salih, Johannes Müller, Ali Eisawi, Faysal Bibi

**Affiliations:** 1 Museum Für Naturkunde, Leibniz Institute for Evolution and Biodiversity Science, Berlin, Germany; 2 Faculty of Petroleum and Minerals, Al Neelain University, Khartoum, Sudan; 3 Department of Geology, University of Khartoum, Khartoum, Sudan

**Keywords:** Palaeontology, Palaeontology

## Abstract

While *Crocodylus* fossils are common in late Cenozoic deposits of Africa, there is a lack of knowledge about species diversity within the genus, especially after the Early Pleistocene. Here we report on a complete skull of a new fossil *Crocodylus* from the Late Pleistocene of the Middle Atbara River, eastern Sudan. Cranial morphology resembles Plio-Pleistocene species of *Crocodylus* from Africa in having upturned squamosals, though not as prominently developed as in these species, whereas the skull differs from fossil and extant *Crocodylus* in having a vaulted sagittal boss on the dorsal surface of the rostrum, and in the absence of a supraoccipital exposure on the dorsal skull table. Phylogenetic analyses indicate the Atbara *Crocodylus* represents a separate species and is more closely related to the fossil African crocodiles than the extant forms. The new species represents the first fossil *Crocodylus* to be described from the Late Pleistocene of Africa, providing new information on the occurrences and diversification of the genus *Crocodylus* during the Late Pleistocene.

**Supplementary Information:**

The online version contains supplementary material available at 10.1038/s41598-025-08980-6.

## Introduction

*Crocodylus* is among the top predators and most widespread genera of the subfamily Crocodylinae, the true crocodiles, with a fossil record that extends back to the Miocene^1^. The genus includes up to 14 extant species that inhabit tropical and subtropical regions of Africa, the Americas, and the Indo-Pacific region, and 5 extinct species, though the validity of some of these taxa remains under discussion. Among extant *Crocodylus*, two species inhabit Africa today: *C. niloticus* and *C. suchus*. Recent molecular studies have demonstrated that the traditionally recognized species *Crocodylus niloticus* actually consists of two genetically distinct lineages, namely *C. niloticus* and *C. suchus*^2–7^. However, distinguishing these two species based on morphology alone has proven challenging^8,9^. *Crocodylus niloticus* is primarily found in eastern and southern Africa, while *C. suchus* occurs mainly in western and central Africa. However, the historical ranges of these two species overlapped considerably^2–10^.

In addition to the extant forms, African fossil *Crocodylus* are known from the Miocene through the Plio-Pleistocene including the Miocene *C. checchiai* (As Sahabi, Libya^1^), the Pleistocene *C. anthropophagus* (Olduvai, Tanzania^11^), and the Plio-Pleistocene *C. thorbjarnarsoni* (Lake Turkana Basin, Kenya^12^). The diversity and evolutionary relationships of these fossil forms remain poorly resolved, particularly given the limited and sometimes unstudied material from the Early Pleistocene onward. Continued research is needed to clarify the biogeographic history, and taxonomic diversity of fossil African *Crocodylus*, and insight into the paleodiversity and dispersal patterns of *Crocodylus* and their relationships to modern African lineages.

Here, we describe a newly discovered fossil cranium from Late Pleistocene sediments in eastern Sudan, which is referable to the genus *Crocodylus*. We establish a new species, compare it with previously known taxa, and present a phylogenetic analysis demonstrating that, despite its relatively recent age, the new taxon represents the youngest known record of an extinct African *Crocodylus*, highlighting its significance as a late-surviving lineage within the clade.

### Geological settings

**Fig. 1:**
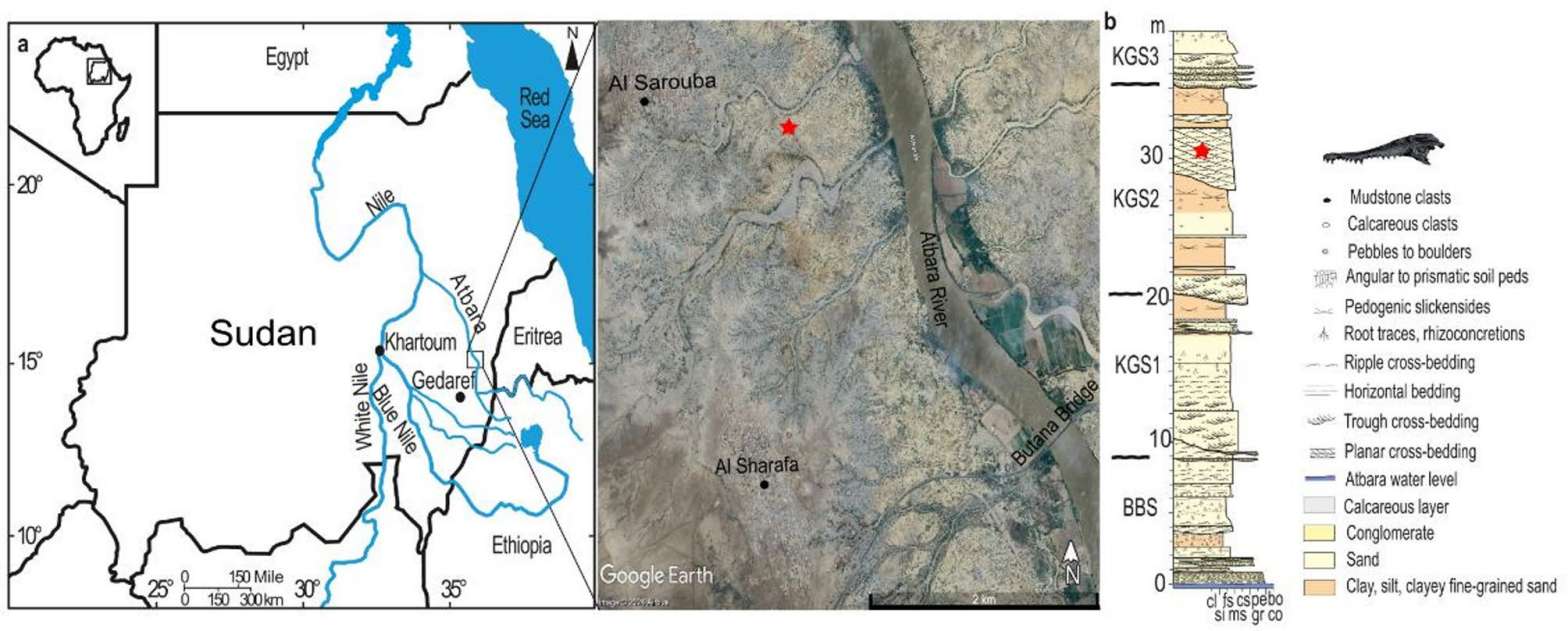
Location map (**a**), and composite stratigraphic profile (**b**) of the Al Sharafa area showing the unit the specimen was recovered from (modified after Mohammednoor et al. 2024^16^). Satellite image from Google Earth (7.3, https://www.google.com/earth/). Figure generated in Inkscape v. 1.3.2. [Full page width].

The fossil-bearing locality where the specimen was found is situated near the Atbara River, close to Al Sharafa village north of Khashm El Girba in eastern Sudan (Fig. 1). The area has long been known to have Pleistocene fossils and artifacts^13,14^. Pleistocene alluvial sediments exposed along the banks of the Atbara and Setit rivers comprise sands and gravels representing channel fills alternating with silt and clay floodplain deposits. Abbate et al.^14^ identified two major units, the Butana Bridge Synthem (BBS) and the overlying Khashm El Girba Synthem (KGS), with each of these further subdivided into three units (BBS 1–3 and KGS1-3). Abbate et al.^14^ dated the BBS as late Early to early Middle Pleistocene (~ 800 ka) based on mammalian biochronology and magnetostratigraphy. U/Th dating of fossil Etheria shells gave an age of 126.1 ± 1.0 ka for the base of the KGS2, and 92.2 ± 0.7 ka for the base of the KGS3. However, luminescence dating by Tsukamoto et al.^15^ revealed a younger and more continuous chronology, with ages of ~ 220–160, ~ 160–130, ~ 130–30, and ~ 30 to < 17 ka for the BBS, KGS1, KGS2, and KGS3, respectively (see also age model in Mohammednoor et al.^16^). This covers Marine Isotope Stages (MIS) 7–2. The paleoenvironment was characterized by humid environments during the deposition of KGS2 with moist grassy habitats adjacent to persistent water and wooded grassland savanna habitats^16^. This suggests a favorable habitat for one or more species of crocodile. The unit where the specimen was collected roughly correlates to the thick sandy channel at around 28–33 m in the composite stratigraphic section of Tsukamoto et al.^15^, which yielded an optical luminescence age of 92 ± 8, and for which the age model of Mohammednoor et al.^16^ indicated an age between ~ 60 and 90 ka (MIS 4–5).

### Institutional abbreviations

**MUST**, Museo Universitario di Scienze della Terra, Sapienza University of Rome, Italy; **sn**, senza numero d’inventario (without inventory number) stored at MUST. **ZMB R**, Reptile Collection, Museum für Naturkunde Berlin, Germany (formerly Zoologisches Museum der Humboldt-Universität zu Berlin).); **MNHN**, Museum Nationale d’Histoire Naturelle (Paris, France); **BMNH**, Natural History Museum, London, formerly known as the British Museum of Natural History.

### Terminology and anatomical abbreviations

Teeth and alveoli are referred to by number, starting with the most anterior. Premaxillary teeth and alveoli have the initials ‘p’, maxillary teeth and alveoli ‘m’, and dentary teeth and alveoli ‘d’. **bo**, basioccipital; **cr**, caecal recesses on wall of caviconchal recess; **ect**, ectopterygoid; **en**, external naris; **eo**, exoccipital; **eoa**, external otic aperture; **f**, frontal; **fm**, foramen magnum; **if**, incisive foramen; **itf**, infratemporal fenestra; **j**, jugal; **l**, lacrimal; **m**, maxilla; **n**, nasal; **o**, orbit; **oc**, occipital condyle; **pa**, parietal; **pal**, palatine; **pf**, prefrontal; **pm**, premaxilla; **po**, postorbital; **pt**, pterygoid; **ptf**, post-temporal fenestra; **q**, quadrate; **qj**, quadratojugal; **soc**, supraoccipital; **sof**, suborbital fenestra; **sq**, squamosal; **stf**, supratemporal fenestra.

## Results

### Systematic paleontology

**Eusuchia** Huxley 1875^17^.

**Crocodylia** Gmelin 1789^18^, sensu Benton and Clark 1988^19^.

**Crocodylidae Gray** 1825^20^.

***Crocodylus*** Laurenti, 1769^21^.

**Fig. 2:**
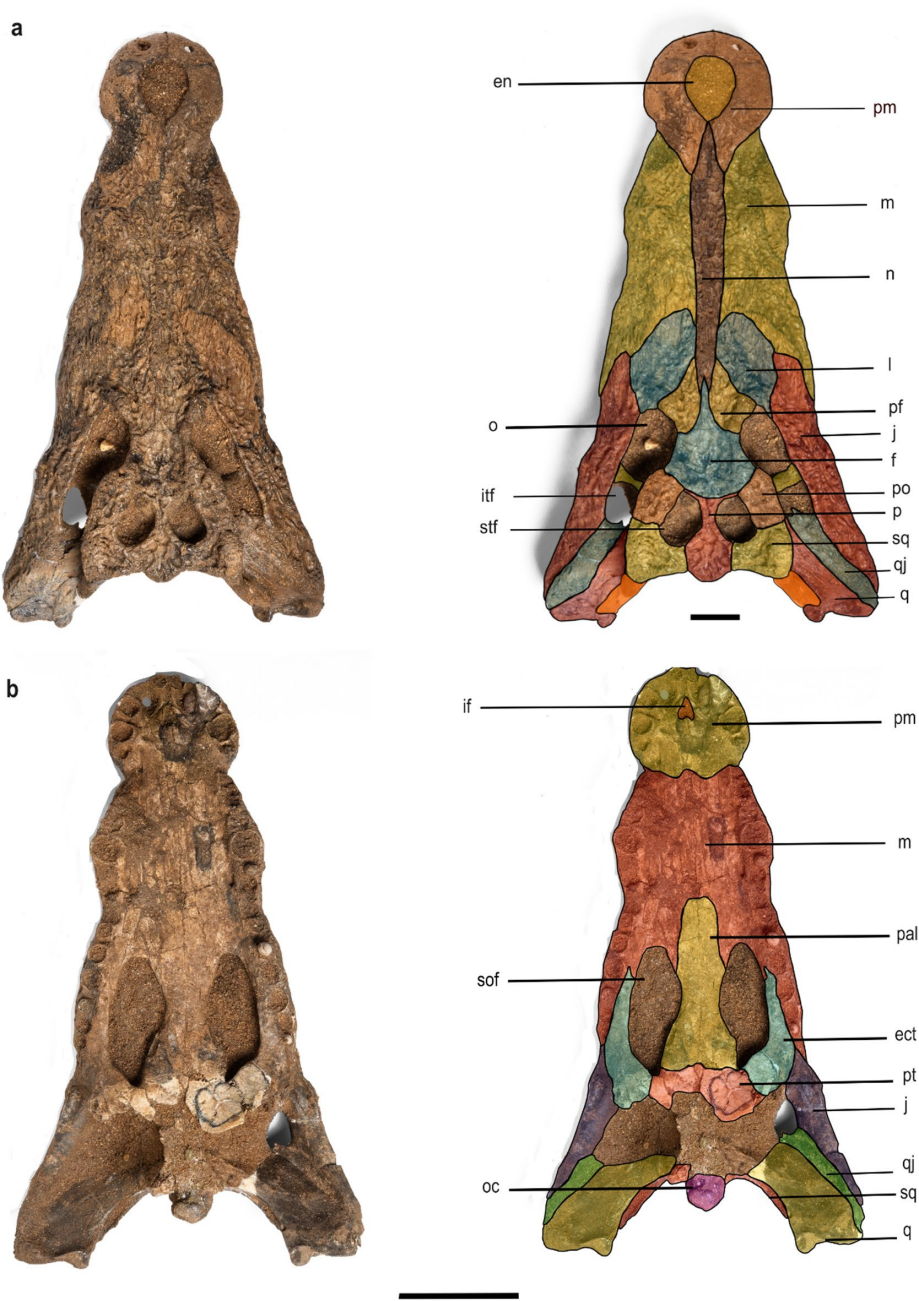
*Crocodylus sudani* holotype cranium, Atbara 22–172, in dorsal (**a**) and ventral (**b**) views. Scale bar: 10 cm. [Full page width].

**Fig. 3:**
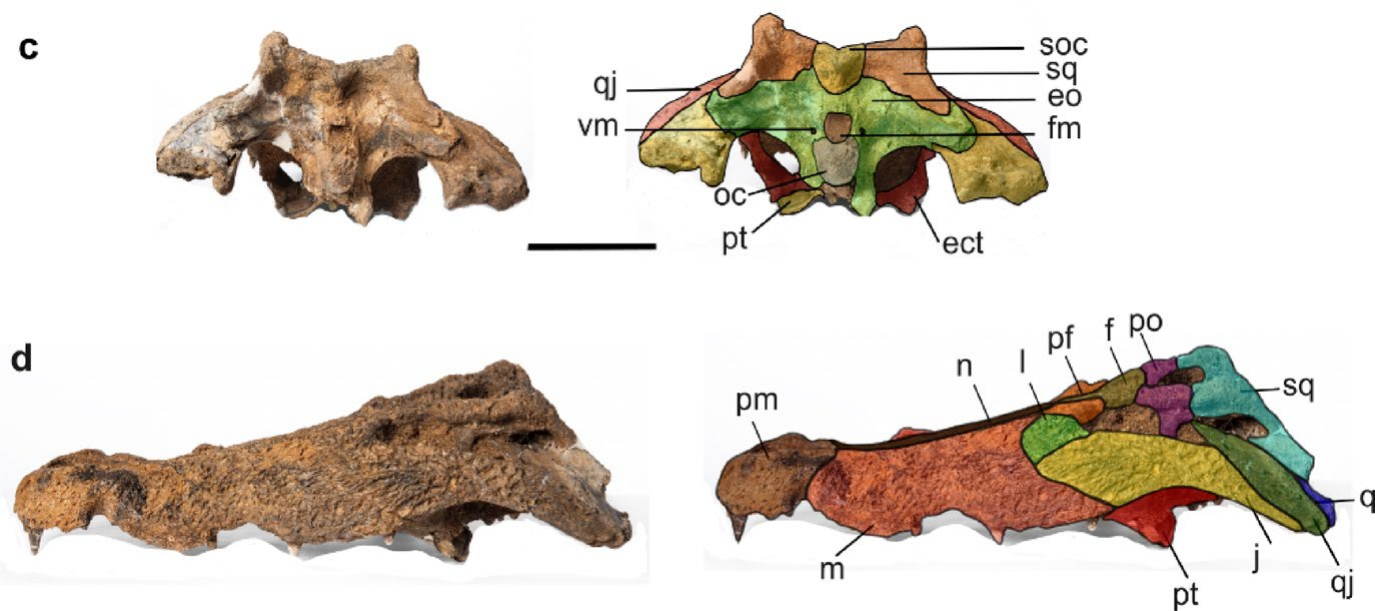
*Crocodylus sudani* holotype cranium, Atbara 22–172, in occipital (**c**) and left lateral (**d**) views. Scale bar: 10 cm. [Half page width].

***Crocodylus sudani, sp. nov.*** (Figs. 2 and 3).

### Holotype specimen

Atbara 22–172, an almost complete skull (Figs. 2 and 3), currently on loan to the Museum für Naturkunde, Berlin, Germany.

### Etymology

The specific epithet is derived from Arabic, the adjective of Sudan, where the material originated.

### Age

Late Pleistocene, from the middle part of the KGS2 subsynthem, excavated from a channel unit with a luminescence age of 92 ± 8 ka (Tsukamoto et al.^15^), or around 90–60 ka based on the age model of Mohammednoor et al.^16^.

### Locality

Found at 15º 5′ 5.8″ N, 35º 56′ 50.0″ E, near the villages of Al Sarouba and Al Sharafa, Gedaref State, Sudan (Fig. 1).

### Diagnosis

A species of *Crocodylus*, characterized by a combination of plesiomorphic and derived features, sharing several traits with the fossil African species *C. checchiai*, *C. thorbjarnarsoni*, and *C. anthropophagus* that distinguish it from *C. niloticus* and *C. suchus*. These include: quadratojugals reaching the posterodorsal corner of the infratemporal fenestra, an anterodorsally oriented external naris, an unforked anterior ramus of the ectopterygoids, prominent prefrontal knobs, and upturned squamosals. *Crocodylus sudani* can be distinguished from *C. anthropophagus* and *C. thorbjarnarsoni* by its relatively narrower snout, a shallower palate, a small but vaulted sagittal boss on the dorsal surface of the rostrum, and the absence of supraoccipital exposure on the skull table. Additionally, it lacks the nasal-maxilla crest found in *C. anthropophagus*.

## Description

### Cranial openings

**The external naris** is teardrop-shaped and anterodorsally directed. It is surrounded by premaxillae, and the nasal enters the posterior narial rim as a pair of thin processes. **The incisive foramen** is heart-shaped, relatively small, and surrounded by premaxillae. It is filled with matrix, making its detailed structures largely invisible through the external naris in the dorsal view. **The orbits** are sub-rectangular dorsally, nearly circular ventrally, and open laterally. Most of the orbital rims are upturned and bordered by the frontal medially, the jugals laterally, the prefrontals anteromedially, the lacrimals anteriorly, and the postorbitals posteriorly. **The supratemporal fenestrae** are D-shaped with upturned medial margins, smaller than the orbits, and bounded by the postorbitals anterolaterally, the parietal anteromedially and medially and squamosals posterolaterally. Both fenestrae are separated medially by the interfenestral bar formed by the parietal. **The infratemporal fenestrae** are triangular, bounded by the postorbitals anteriorly, the quadratojugals posteriorly, the squamosals medially, and the jugals ventrolaterally. **The external otic aperture** is leaf-shaped and surrounded partially by the squamosals posterodorsally and by the quadrates ventrally. The quadrato–squamosal suture extends dorsally along the posterior margin of the aperture. **The suborbital fenestrae** are anteroposteriorly oval and bounded by the maxillae anteriorly, the ectopterygoids posterolaterally, the pterygoid posteromedially, and the palatine medially. They extend anteriorly to m10. **The foramen magnum** is filled with matrix and surrounded by the exoccipitals dorsally. The basioccipital forms the occipital condyle and bounds the foramen ventrally. **The post-temporal fenestrae** are largely obliterated by the matrix but appear to be small openings on the occipital surface of the skull. They are surrounded dorsolaterally by the squamosals, and ventrally and dorsomedially by the exoccipital.

### Cranial elements

**The premaxillae** are well preserved with shallowly pitted dorsal ornamentation. Close to the anterior tip of the snout, each premaxilla is perforated by an opening for the occlusion of the d1. The premaxillary-maxillary suture is at the level of the p4 alveoli with a notable constriction for reception of the d4. In dorsal view, an acute process extends posteriorly to the level of the m2. In ventral view, the premaxillary-maxillary suture is convex, having a broad W-shape at the level of the m2. Each premaxilla possesses five alveoli, with the fourth being the largest. There is a highly-vaulted occlusal pit, which is perforated on both sides between p1 and p2 for hosting the d1.

**Fig. 4:**
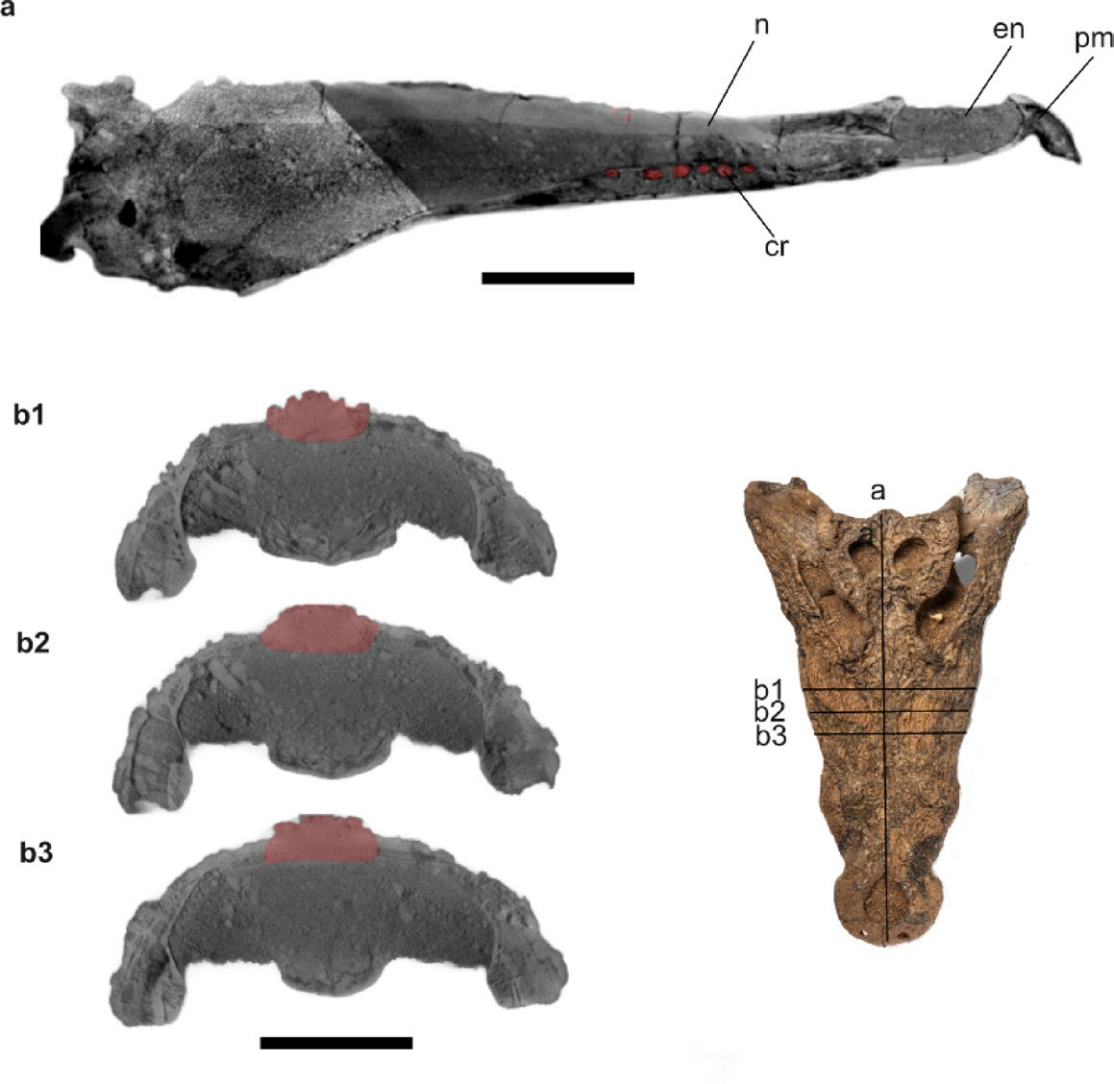
Atbara 22–172 cranium, CT images showing the shallow pits within the medial wall of the caviconchal recess in red (**a**), and the vaulted sagittal boss on the dorsal surface of the rostrum in red (**b1**-**b3**). Scale bar: 10 cm. [Full page width].

**The maxillae** are long and broad, dorsoventrally deep, and laterally ornamented with shallow pits. In dorsal view, they are separated by the nasal medially via a sharp linear crest parallel to the maxilla-nasal suture. They are bounded by the jugal posterolaterally and the lacrimals posteromedially. Dorsally, the maxillae bear a prominent circular protuberance dorsal to the m5. The lateral maxillary margins are undulating with a notable constriction at the level between m8 and m9. Medially, the maxillae preserve a linear array of shallow pits within the medial wall of the caviconchal recess (Fig. 4). Ventrally, the maxillae are vaulted, contacting the palatine at the level of m8 and the ectopterygoids at the level of m11. Each maxilla has fourteen alveoli with the fifth being the largest. Diastemata separate the m9 and m10 alveoli; the remaining alveoli are close together. Deep occlusion pits occur between m8 and m9. **The nasal** is long and narrow, placed between the maxillae, and ornamented with shallow pits on its dorsal surface. It tapers anteriorly when it contacts the external naris, and posteriorly when it contacts the lacrimals, prefrontals, and frontal.

**The prefrontals** are butterfly-shaped, forming the anteromedial margins of the orbits. They are separated from each other by the anterior process of the frontal posteriorly and the nasal anteriorly. The prefrontals extend more anteriorly than the frontal, and their contact with the lacrimals is linear in shape. Along the orbits’ margins, the bones possess two distinct knobs.

**The lacrimals** are leaf-shaped and extend further anteriorly than the prefrontals, forming much of the anterior margin of the orbits. Dorsally, the bones are ornamented with shallow furrows. The lacrimals contact the jugals laterally, the maxillae anteriorly, the prefrontals posteromedially, and the nasal medially. The contact with the prefrontals is almost three times as extensive as the contact with the nasals. The lacrimals possess thin anteroposteriorly oriented crests on their dorsal surfaces extending from the lacrimal-prefrontal suture at the orbital margins.

**The jugals** are elongated and laterally ornamented with shallow furrows anteriorly, and with pits posteriorly. The anterior ramus of the jugals extends to contact the maxillae anteriorly and the lacrimals medially, and the jugals also form the lateral margins of the orbits. Also, the jugals form the ventral half of the postorbital bar. Dorsoventrally, the jugals become relatively thin and taper posteriorly. The posterior rami are flattened mediolaterally and form the ventral margins and the posteroventral angle of the infratemporal fenestrae.

**The frontal** is a single broad bone that bears an acute anterior process between the prefrontals reaching the nasal anteriorly. The bone contacts the parietal posteriorly and the postorbitals posterolaterally in a convex suture. The lateral margins are upturned along the orbits, whereas the dorsal surface is flat and ornamented with shallow grooves, without a mid-sagittal crest. Ventrally, the frontal possesses a deep groove for the olfactory tract. **The postorbitals** are crescent-shaped and form the posterolateral margin of the orbit and the anterolateral margin of the supratemporal fenestrae. They extend posteriorly to about two-thirds the length of the supratemporal fenestrae to contact the squamosals. They are ornamented dorsally with pits. Ventrally, each postorbital sends a descending process to form the dorsal half of the postorbital bar. On the lateral surface, there is a deep groove, as well as a vascular foramen dorsal to the postorbital bar.

**Fig. 5:**
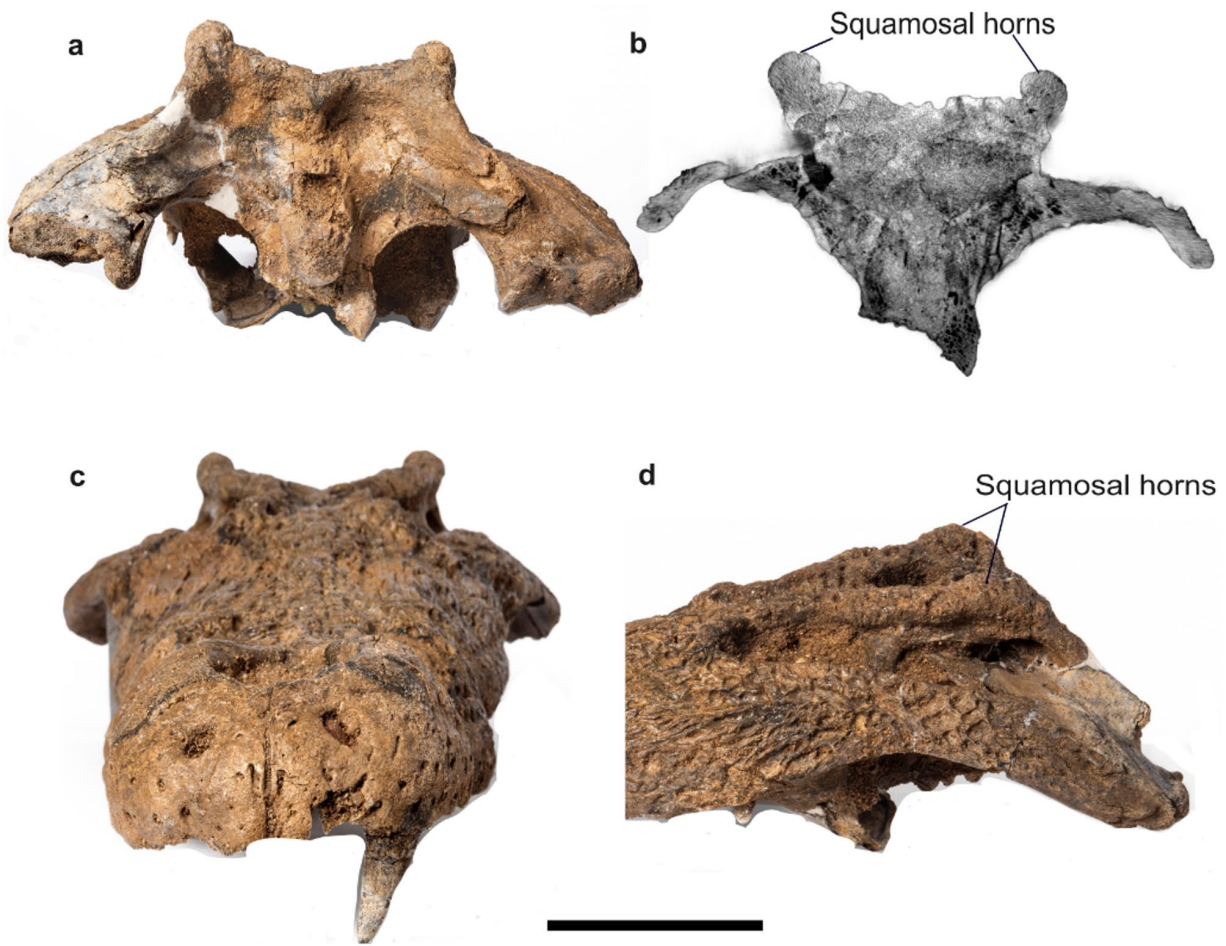
Atbara 22–172 cranium highlighting the squamosal horns in occipital view (**a**), a CT transversal section through the horns (**b**), anterior view (**c**), and right lateral view (**d**). Scale bar: 10 cm. [Full page width].

**The parietal** is completely fused, narrows anteriorly, and widens posteriorly. Its dorsal surface is sculptured with shallow grooves. The bone forms the medial margin of the supratemporal fenestrae with upturned lateral margins and contacts the frontal anteriorly, the supraoccipital posteriorly, and the squamosals laterally at the mid-section of the posterior wall of the supratemporal fenestrae. The posterior margin of the parietal, and thus of the skull roof, tapers posteriorly. **The squamosals** form the posterolateral margin of the supratemporal fenestra and represent one-third of the lateral margins of the supratemporal fenestrae. They contact the postorbital anteriorly, the parietal medially, and the quadrate posteroventrally. The lateral surfaces of the squamosals are oriented anteromedially with a relatively high angle between them and the sagittal axis, resulting in a trapezoidal shape of the skull table. The dorsal surface of the squamosals is upraised, forming a low crest along its lateral margin with a well-developed horn-like element immediately dorsal to the otic aperture, forming a gentle U-shaped skull table in occipital view (Fig. 5). In lateral view, this element is sub-triangular in shape and rounded dorsally, with a steeper posterior margin. The squamosals bear a lateral groove continuous with the lateral surface of the postorbital for attachment of the ear flap musculature. In occipital view, the squamosals contribute to the post-temporal fenestra and extend ventrolaterally to the lateral extent of the paraoccipital process of the exoccipitals. A descending lamina of the squamosals laps onto the dorsal surface of the quadrate ramus.

**The quadratojugals** are situated between the quadrates and the jugals, extending posteromedially to the lateral quadrate hemicondyles. The quadratojugals form the posterior margin of the infratemporal fenestra and send a short spine-like process into the fenestra. **The quadrates** are triangular, contacting the squamosals dorsally and forming the posterior margin of the otic aperture. The quadrates extend anteriorly to contact the postorbitals at a high angle. They extend posteriorly to form rami bearing the small foramen aëreum on their dorsomedial surface. In occipital view, the cranioquadrate canal opens between the quadrate and the paraoccipital process of the exoccipital lateral to the occipital condyle. The quadrates form the dorsal margin of the partially preserved basioccipital. The medial hemicondyle is larger and exhibits greater dorsoventral expansion compared to the lateral hemicondyle.

**The palatine** is long and anteriorly forms a U-shaped anterior process with a convex margin where it contacts the maxillae. Posteriorly, it contacts the pterygoid at the level of the posterior margin of the suborbital fenestrae, its contact with the suborbital fenestrae being concave. **The ectopterygoids** are partially preserved and form the medial margin of the posteriormost maxillary alveoli as well as the lateral margin of the suborbital fenestrae. They contact the maxillae anteriorly at the level of m11. Their anterior tip is unforked. The ectopterygoids contact the pterygoid medially in a linear suture at the posterior margin of the suborbital fenestra. Posteriorly, their relationship with the pterygoidal ramus and the pterygoid wings is unknown due to poor preservation.

Only the anterior portion of the pterygoids are preserved, which articulate with the palatines anteriorly in a broad, W-shaped suture. They form the posteromedial margin of the suborbital fenestrae. Nothing else can be described because the posterior part of the pterygoid including the choanae as well as the pterygoid wings is missing. **The supraoccipital** is wide and V-shaped. It is dorsally bounded by the parietal and ventrally by the exoccipitals, which separates it from the foramen magnum. The supraoccipital does not seem to contribute to the post-temporal fenestrae. It shows a median crest for the attachment of the cervical muscles.

**The exoccipitals** are wide, meet dorsomedial to the foramen magnum, and extend laterally to form the paraoccipital processes. Ventrally, they contact the quadrates at the position where the cranioquadrate canal passes. The exoccipitals contact the squamosals dorsolaterally and the supraoccipital dorsomedially. The vagus foramen is visible lateral to the occipital condyle on both sides and opens within a shallow depression. **The basioccipital** is partially preserved and since most of the bone is broken and missing, it is impossible to evaluate its ventral extension, including the relationship with the basisphenoid and the arrangement of the Eustachian foramina.

### Anatomical comparisons

**Fig. 6:**
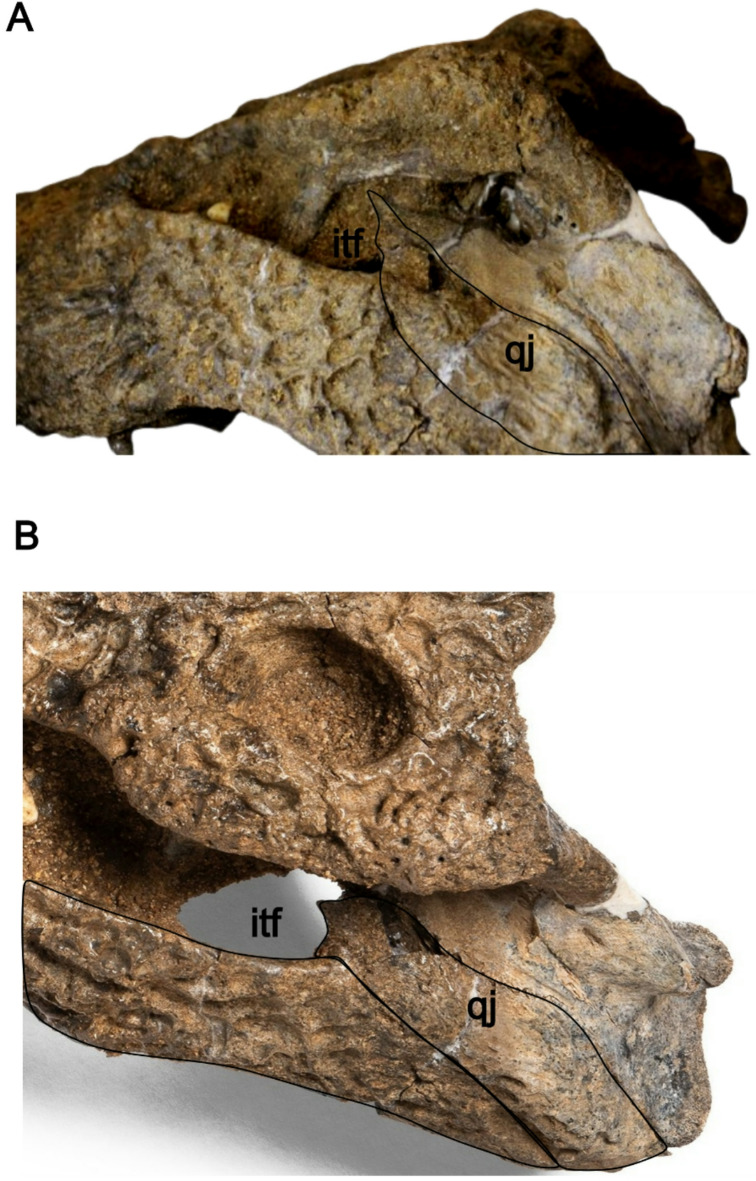
Atbara 22–172 cranium showing the extension of the quadratojugal to the dorsal corner of the infratemporal fenestra in dorsal (**a**) and left lateral (**b**) view. [Full page width].

**Fig. 7:**
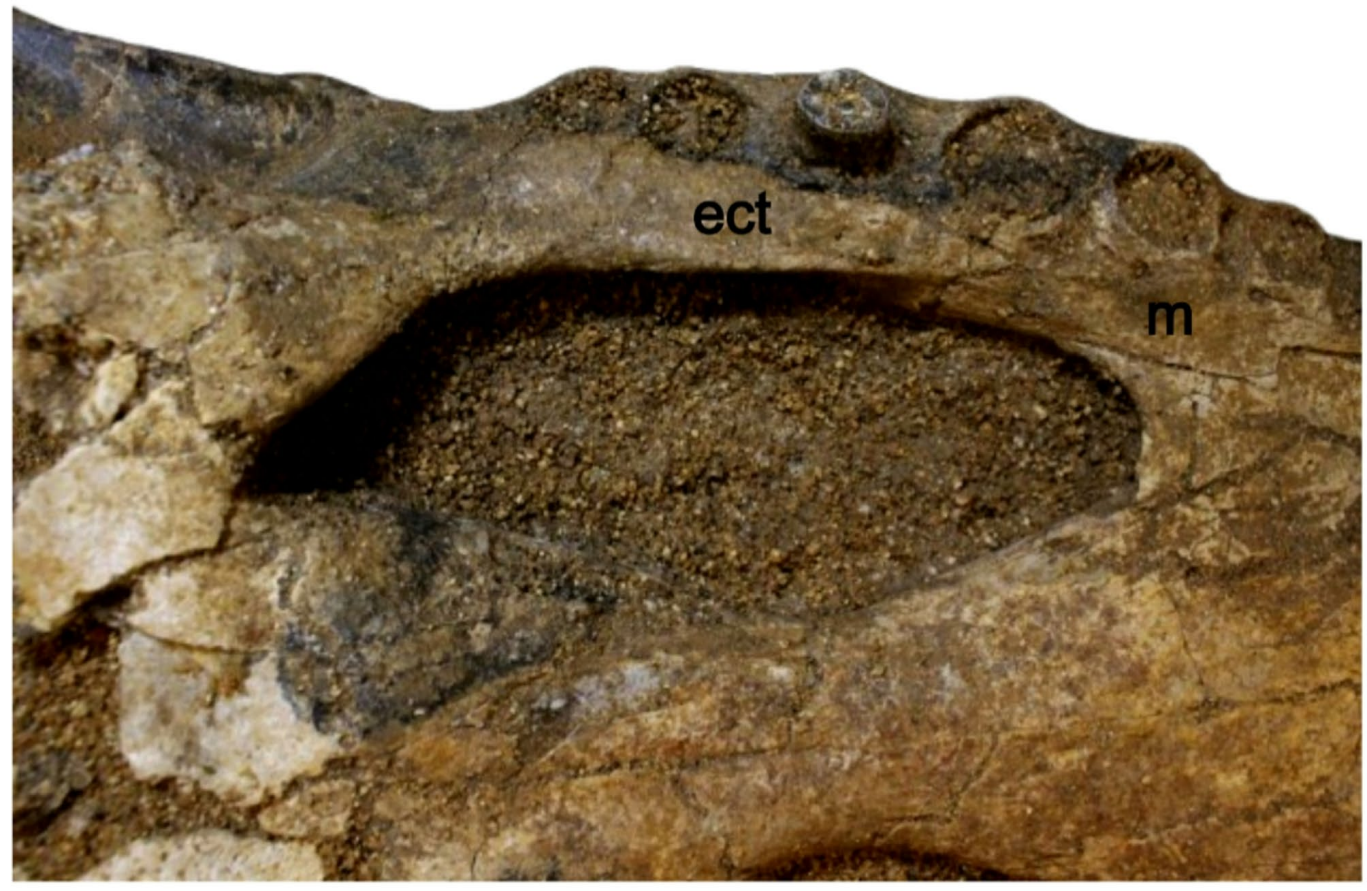
Atbara 22–172 cranium showing unforked anterior ramus of the ectopterygoids in ventral view. [Half page width].

Due to the considerable morphological similarities between the skulls of *C. niloticus* and *C. suchus*, distinguishing the two species based solely on skull morphology alone is difficult. As a result, we refer to museum specimens assigned to the common African crocodile collectively as *C. niloticus*/*suchus*. The presence of a linear array of shallow pits within the medial wall of the caviconchal recess supports the attribution of Atbara 22–172 to the genus *Crocodylus*^12^. The general morphology of Atbara 22–172 resembles superficially *C. niloticus/suchus* (e.g. in snout shape), but several characters are clearly different and exclude it from these extant species. The external naris is anterodorsally oriented, and the skull table is trapezoidal with an angle of 11° between the lateral margin of the skull table and the sagittal axis. In contrast, *C. niloticus*/*suchus* has a rectangular skull table and the external naris is dorsally oriented. Also, Atbara 22–172 has a pair of prominent knobs on the prefrontals as well as several prominent dorsal projections at the posterolateral corner of the squamosals, i.e. features that are normally absent or only occasionally found, but relatively less developed in some very large individuals of *C. niloticus/suchus* (Brochu^11^ and personal observation). In Atbara 22–172, the quadratojugals form the posterodorsal corner of the infratemporal fenestra (Fig. 6), while it is formed by the quadrate in *C. niloticus/suchus*^12,22^. Finally, the anterior maxillary process of the ectopterygoid in this specimen is unforked (Fig. 7), a condition that is “variably expressed” in extant African *Crocodylus*^12^ and is less common than the more frequently observed forked morphology^15^. Moreover, we compared the skull proportions of Atbara 22–172 with closely similar-sized *C. niloticus/suchus* specimens (Table 1), all with skull lengths ranging from 46 to 60 cm. The snout/skull length ratio of Atbara 22–172 is 66.67, similar to that of *C. niloticus*/*suchus* at 68.43 ± 3.02 (mean ± 2 standard deviations). The skull width/length ratios at the quadrates and m5 are 59.65 and 33.33, respectively in Atbara 22–172, and 57.63 ± 9.48 and 33.14 ± 6.24 in *C. niloticus*/*suchus.* Thus, Atbara 22–172 appears to be similar in length to those of *C. niloticus/suchus.* However, this comparison should be taken with caution considering the ontogenetic and ecophenotypic variation in the extant forms^23^.

**Table 1:** Skull measurements of Atbara 22–172 compared to other *Crocodylus niloticus*/*suchus* specimens.

Specimen No	SkL	SnL	Sn/Sk	SkWq	SkWq/SkL	Skm5	SkWm5/SkL
ZMB R 36,650	48.5	33.1	68.25	28	57.73	15.8	32.58
ZMB R 17,913	50.7	35.2	69.43	31.4	61.93	19.1	37.67
ZMB R 36,655	52.7	34.8	66.034	33.3	63.19	20.5	38.90
ZMB R 36,656	57.3	38.6	67.36	34.9	60.91	21.8	38.05
MNHN 1996–8280	62	43	69.35	38	61.29	21	33.87
BMNH 1975.1313	58	41	70.69	33	56.90	18	31.03
BMNH 1900.9.22.2	48	31	64.58	25	52.08	15	31.25
BMNH 1949.1.1.2	60	41	68.33	36	60.00	19	31.67
BMNH 1911.8.15.1	51	35	68.63	29	56.86	16	31.37
BMNH 1934.6.3.1	50	34	68.00	27	54.00	15	30.00
BMNH 1865.4.6.2	55	38	69.09	31	56.36	18	32.73
BMNH 1865.5.3.76	50	35	70.00	29	58.00	15.5	31.00
BMNH 1967.1074	51	34.5	67.65	32	62.75	20	39.22
BMNH 1967.1075	55.5	39	70.27	32	57.66	18	32.43
BMNH 1967.1076	59	41	69.49	36	61.02	18.5	31.36
BMNH 1959.1.8.55	46	32	69.57	25.5	55.43	15	32.61
BMNH 1897.6.24.1	48	33	68.75	27.5	57.29	14	29.17
BMNH 1862.6.30.7	53.5	37	69.16	32	59.81	19	35.51
BMNH 1969.1565	55	37	67.27	23	41.82	16	29.09
Atbara 22–172	57	38	66.67	34	59.65	19	33.33

SkL, Skull length; SnL, Snout length measured from the anterior orbit to the tip of the premaxilla; SkWq, Skull width at quadrates; SkWm5, Skull width at maxillary fifth tooth.

Atbara 22–172 is similar to *C. thorbjarnarsoni* and *C. anthropophagus* in having an anterodorsal orientation of the external naris; a trapezoidal skull table (the angle is about 11˚ between the lateral margin of the skull table and the sagittal axis in *C. thorbjarnarsoni*); quadratojugals forming the posterodorsal corner of the infratemporal fenestra; an unforked anterior process of the ectopterygoid; a pair of prominent knobs on the prefrontals; and the presence of dorsal projections at the posterolateral corner of the squamosals, although not as prominent as in *C. anthropophagus*. Compared to *C. thorbjarnarsoni*, Atbara 22–172 has a relatively smaller skull and body size (skull length is 85 cm in *C. thorbjarnarsoni* and 56 cm in Atbara 22–172), as well as a narrower and less deep snout. The posterior margin of the parietal and thus of the skull roof, tapers posteriorly (straight in *C. thorbjarnarsoni*), in addition to the squamosals reaching substantially onto the dorsal surface of the quadrate ramus, which is not seen in *C. thorbjarnarsoni.* Despite the incomplete material of *C. anthropophagus*, we can exclude the attribution of Atbara 22–172 to this species based on the following characters that characterize *C. anthropophagus*: a pair of prominent dorsal crests on the lacrimal; a pair of prominent thin crests along the maxilla-nasal suture; the anterior end of prefrontal and frontal being at the same level and more prominent triangular dorsal projections at the posterolateral corner of the squamosals. Atbara 22–172 shares a vaulted sagittal boss on the dorsal surface of the rostrum (Fig. 3) but not as seen in the Miocene *Crocodylus checchiai* from Libya (sn813/lj^1^). However, the morphology of the squamosals, the trapezoidal skull table, and the separation of the nasals from the external naris allow for the exclusion of Atbara 22–172 from the geologically older *Crocodylus checchiai*.

### Phylogenetic analysis

**Fig. 8:**
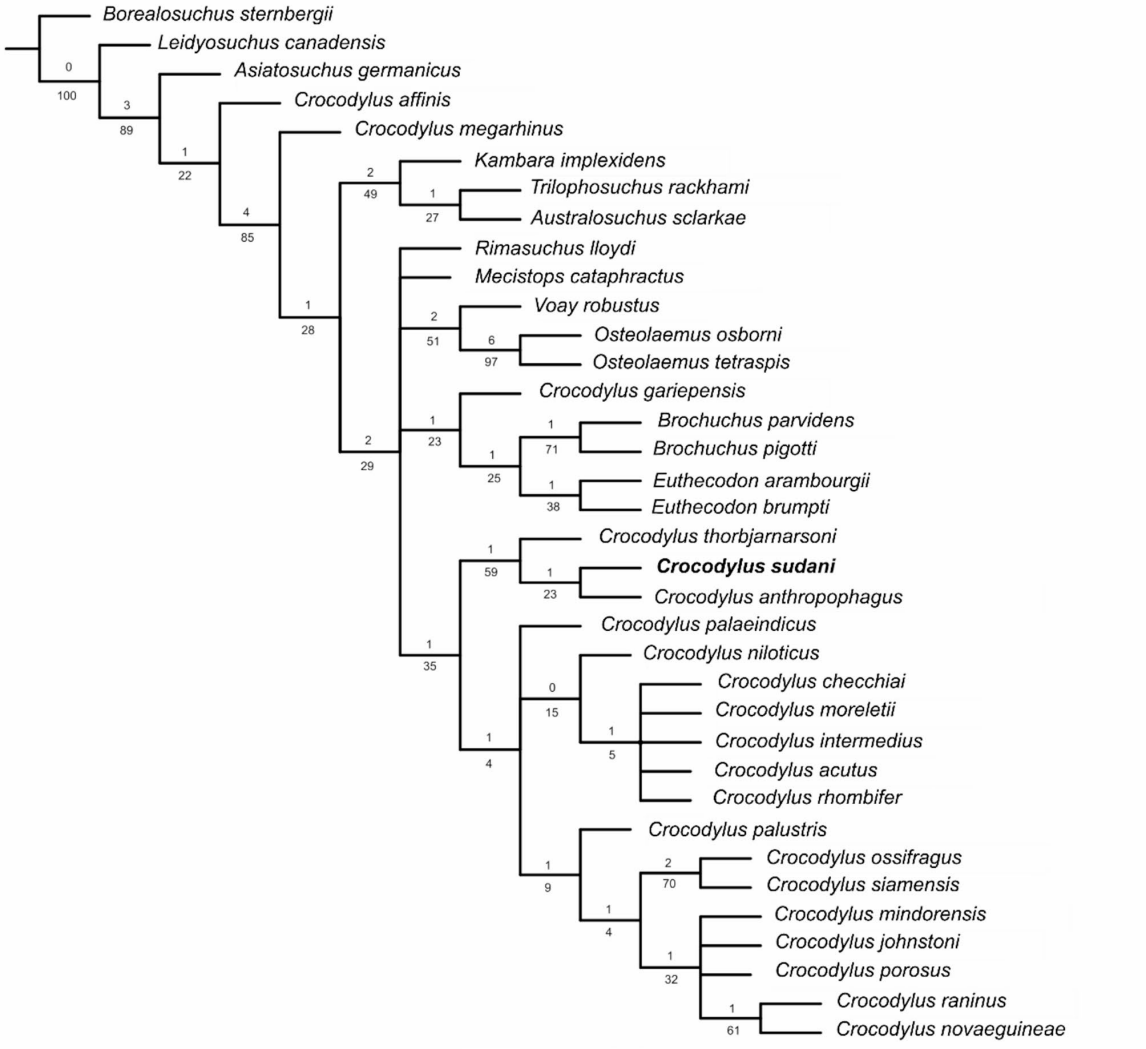
Strict consensus of four equally parsimonious trees showing the position of *Crocodylus sudani* based on the matrix of Azzara et al. (2021)^22^. Tree length = 220 steps, consistency index = 0.586, retention index = 0.710. Branch numbers: bootstrap (bottom) and Bremer support (top). [Half page width].

Using the Azzara et al.^22^ matrix, four equally parsimonious trees were recovered (220 steps, consistency index = 0.586, retention index = 0.710). The strict consensus tree does not differ significantly from Azzara et al.^22^, except for the collapse of the monophyly of Osteolaeminae and Crocodylinae (Fig. 8). Also, *C. niloticus* is basal to a paraphyletic clade including *C. checchiai* + Neotropical *Crocodylus* and more closely related to Neotropical than Indo-Pacific *Crocodylus*. *Crocodylus sudani* is united with *C. thorbjarnarsoni* and *C. anthropophagus* in a clade that is supported by four synapomorphies: an external naris that projects anterodorsally (81:0), prominent prefrontal knobs (107:1), quadratojugals that extend to the posterodorsal corner of the infratemporal fenestra (142:0) and the presence of upturned squamosals (157:1). *Crocodylus anthropophagus* and *C. sudani* are further united by sharing an unforked anterior process of the ectopterygoids and a linear posterolateral margin of the suborbital fenestra (119:0). *Crocodylus sudani* differs from *C. anthropophagus* by having a vaulted sagittal boss on the dorsal surface of the rostrum (95:1) and lacking the supraoccipital exposure on the dorsal skull table (160:1).

**Fig. 9:**
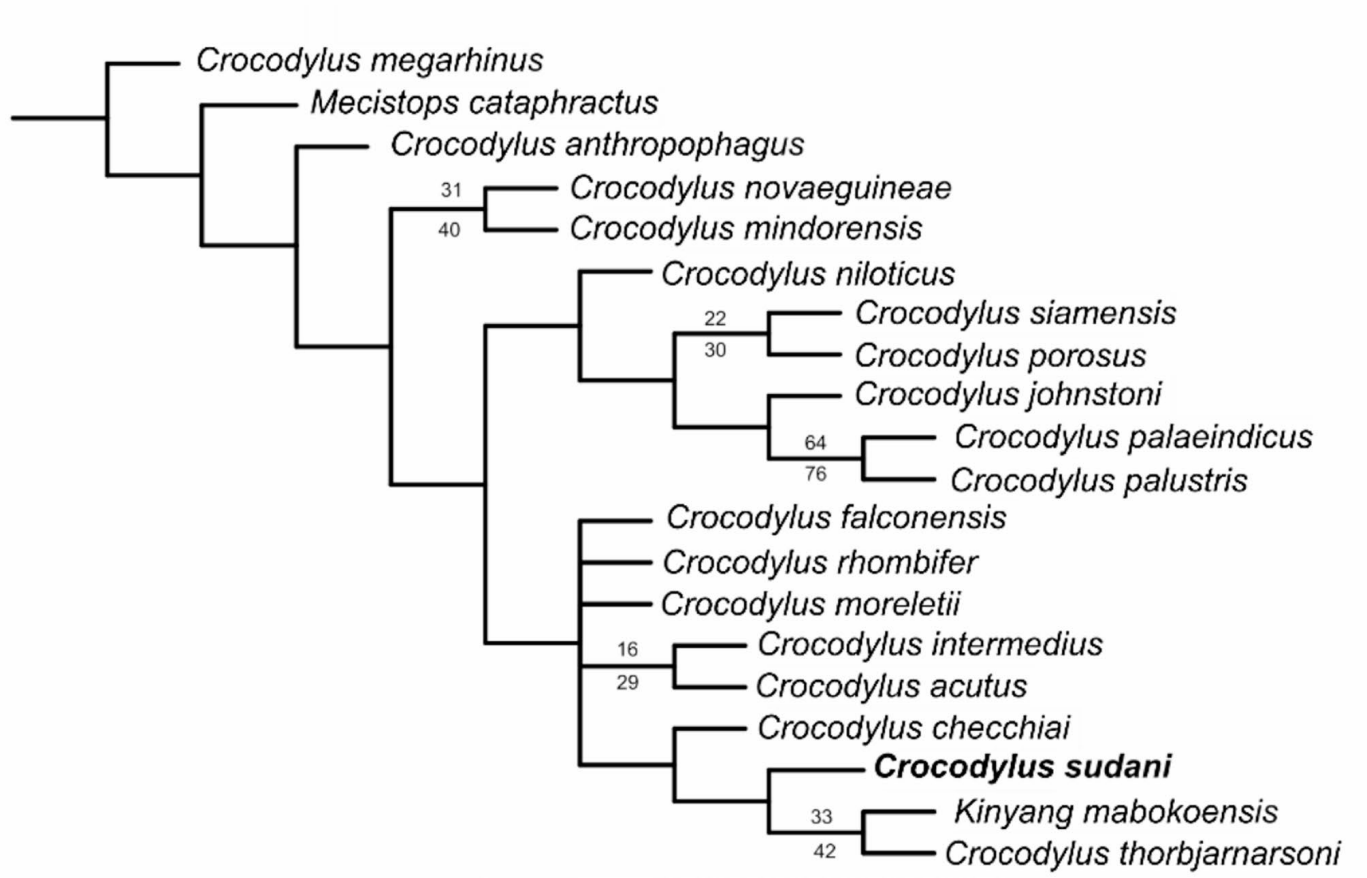
Strict consensus of three equally parsimonious cladograms showing the position of *Crocodylus sudani* based on the matrix of Chabrol et al. (2024)^24^. Branch numbers: bootstrap (top) and jackknife (bottom). [Half page width].

In the phylogenetic framework of Chabrol et al.^24^*, C. sudan*i was recovered as part of a clade of African fossil *Crocodylus* that included *C. thorbjarnarsoni* and *Kinyang mabokoensis,* united by three unambiguous synapomorphies: the mediolateral width to anteroposterior length ratio of the external naris is ≤ 1 (3:0); the position of the foramen aereum on the posterior ramus of the quadrate, located dorsomedially (117:0); and the posterior extension of the quadratojugal conceals the lateral surface of the quadrate condyle (120:0) (Fig. 9). This clade was recovered as the sister group of a clade uniting *C. niloticus* with *C. palustris*.Both analyses show low support values, with most nodes having bootstrap values below 50% and Bremer support values of 0–2, highlighting uncertainty in the phylogenetic relationships within *Crocodylus*. Results from both phylogenetic analyses nonetheless support a closer affiliation of *C. sudani* with other fossil African *Crocodylus*, while rejecting a close relationship with *C. niloticus*/*suchus*.

## Discussion

Our study demonstrates that the cranial morphology of specimen Atbara 22–172 differs from both extant and other extinct African *Crocodylus* species, thereby providing a basis for the establishment of a new species, *Crocodylus sudani*. Moreover, although the new taxon shares some characters with fossil African species such as *C. anthropophagus* and *C. thornbjarsoni* from Kanapoi (e.g. an anterodorsally oriented external naris, prominent prefrontal knobs, upturned squamosals and vaulted sagittal boss on the dorsal surface of the rostrum), *Crocodylus sudani* can be distinguished from *C. anthropophagus* and *C. thorbjarnarsoni* by its relatively narrower snout, a shallower palate, and the absence of supraoccipital exposure on the skull table. Additionally, it lacks the nasal-maxilla crest found in *C. anthropophagus*. Based on the cranial length to total body length ratio^28^, *C. sudani* is estimated to have reached a maximum length of 4.0–4.3 m.

According to Hekkala et al.^3^, *C. suchus* can be found in Ethiopia and Uganda, indicating that it is still widespread throughout East Africa. Diversification within the extant *C. suchus* clade is believed to have occurred during the Mid to Late Pleistocene, due to the aridification and population isolation, at a time when the Niger Delta was connected to the Nile Basin via Mega Lake Chad and the Sudd wetlands in South Sudan^3,26^. Additionally, the historical sympatry of *C. niloticus* and *C. suchus* in Egypt and the upper White Nile in South Sudan has been confirmed by DNA analysis of *Crocodylus* specimens^3,25^. Owing to the superficial morphological similarities between *C. sudani* and *C. niloticus*/*suchus* and the lack of precise sampling, one can argue that *C. sudani* might still live in the Nile Basin today, i.e. as a cryptic species suggesting the presence of a previously unrecognized lineage during the Pleistocene. While these distinctions are currently restricted to the fossil record, the possibility that *C. sudani* represents a lineage that persisted longer or may even persist undetected warrants further investigation. However, a comprehensive and geographically broad sampling of extant *Crocodylus* populations in Sudan and surrounding regions would be required to investigate this hypothesis.

Using two different morphological matrices, our phylogenetic analyses support the distinctiveness of *C. sudani* from *C. niloticus*/*suchus* However, both these datasets have very low internal statistical support, with most nodes having bootstrap values below 50% and Bremer support values of 1–2^11,12,22^. This suggests that a comprehensive review of *Crocodylus* phylogeny is needed, including not only broader taxon and data sampling, but also a re-evaluation of the morphological characters used in previous analyses.

*Crocodylus sudani* is the geologically youngest extinct species of African *Crocodylus* and the first fossil *Crocodylus* to be described from the Late Pleistocene of Africa and indicates the persistence of higher diversity in this clade well into the Quaternary. Owing to the superficial morphological similarities between *Crocodylus sudani* and *C. niloticus*/*suchus,* and the lack of precise sampling, one might consider the possibility that the Atbara *Crocodylus* survived into the historical period, or even that it might continue to inhabit the Nile Basin today.

## Methods

### μCT imaging

The specimen was subjected to micro-tomographic analysis at the Museum für Naturkunde Berlin (Laboratory identification ID RRID: SCR_022585) using a Comet YXLON FF85 (Comet YXLON GmbH, Hamburg, Germany; Equipment identification ID RRID: SCR_020917). The specimen was scanned in 3 vertical scans and stacked together by hand at 300 kV and 700μA, generating 2500 projections with 133 ms per scan. The different kV- and projection settings depend on the respective specimen size, which is also responsible for the effective voxel size at 20 μm. The cone beam reconstruction was performed using the Nexus reconstruction software (Comet YXLON GmbH, Hamburg, Germany) as well as a double horizontal extension of the field of view for each scan. The data were visualized using VG Studio Max 3.5 (Volume Graphics GmbH).

### Phylogenetic analysis

To evaluate the phylogenetic position of the new taxon and explore alternative topologies, phylogenetic analysis was performed employing the matrix of Brochu and Storrs^15^ as modified by Azzarà et al.^26^ as well as the expanded matrix of Chabrol et al.^24^. The Azzara et al. matrix includes 36 taxa and 189 morphological characters, of which 82 characters (44%) could be confidently scored for specimen Atbara 22–172. All characters were treated with equal weights; multistate characters were left unordered. The analysis was conducted using parsimony in TNT v. 1.629, with TNT’s "New Technology Search" algorithm. *Borealosuchus sternbergii* was designated as the outgroup.

The matrix of Chabrol et al.^24^ comprises 155 operational taxonomic units (OTUs) scored for 330 characters, of which 158 (48%) could be scored for Atbara 22–172. Characters used were both discrete and continuous characters, and 36 multistate characters were treated as ordered. 14 topological constraints reflecting molecular phylogenetic relationships were applied following Chabrol et al^24^. *Bernissartia fagesii* was set as the outgroup. For both analyses, bootstrap (10,000 replicates), Bremer support, and Jackknife analyses were performed to evaluate internal statistical support for recovered clades. Nexus files for running both analyses are provided as supplementary materials.

#### Nomenclatural acts

This work and the nomenclatural acts have been registered in the online registration system (ZooBank) for the International Code of Zoological Nomenclature under registration numbers urn:lsid:zoobank.org:pub:075DC5DC-5515-4968-A3EC-0867D1E702B6 and urn:lsid:zoobank.org:act:8C4C617F-0FBC-42C4-889F-83B74556F3F4.

## Electronic supplementary material

Below is the link to the electronic supplementary material.

Supplementary Material 1

Supplementary Material 2

## Data Availability

All data generated or analyzed during this study are included in this published article, its Supplementary Information files, and through Morphosource (https://www.morphosource.org/concern/media/000703568?locale=en).
